# Validation of field-based running tests to determine maximal aerobic speed in professional rugby league

**DOI:** 10.1371/journal.pone.0306062

**Published:** 2024-07-17

**Authors:** Thomas Bennett, Phil Marshall, Steve Barrett, James J. Malone, Andrew Simpson, James Bray, Calum Christopherson, Tom Nickolay, James Metcalfe, Chris Towlson

**Affiliations:** 1 Hull F.C., Hull, United Kingdom; 2 School of Sport, Exercise and Rehabilitation Sciences, University of Hull, Hull, United Kingdom; 3 Playermaker, London, United Kingdom; 4 School of Health and Sport Sciences, Liverpool Hope University, Liverpool, United Kingdom; University of Pavia: Universita degli Studi di Pavia, ITALY

## Abstract

Practitioners place importance on high-speed running (HSR) to monitor training practice and match-play demands, whilst attempting to maximise fitness and minimize the risk of injury occurrence. Practitioners apply various methods to quantify HSR, such as absolute thresholds, percentage of maximum sprint speed and maximal aerobic speed (MAS). A recent survey demonstrates the 5-minute run and 1200m shuttle test (ST) to be implemented among rugby league practitioners to quantify HSR by incorporating MAS. However, it is unclear as to how valid these methods are to accurately quantify MAS. Therefore, the aim of this study was to assess the validity of the 5-minute run and 1200m ST when compared to a gold standard measure for MAS. Twenty 1^st^ team professional rugby league players competing in the European Super League participated in this study. Players were required to complete an incremental treadmill test, 5-minute run and 1200m ST over a two-week period in pre-season. MAS, peak heart rate (HR_max_), peak lactate (La_peak_) and rating of perceived exertion (RPE) where collected upon completion of each test. Results demonstrated the 1200m ST to have a higher correlation for MAS than the 5-minute run (1200m ST: r = 0.73, 5-minute run: r = 0.64). However, when assessing validity using the level of agreement between data, the 5-minute run underreported MAS by 0.45 m·s^-1^ whereas the 1200m ST underreported MAS by 0.77 m·s^-1^. Ultimately, both field-based tests used in this study underreport MAS when compared to an incremental treadmill test, although the 5-minute run provides a closer agreement and therefore a more valid measurement for MAS than the 1200m ST.

## Introduction

Quantifying the physical outputs of rugby league is becoming common practice, with practitioners utilising metrics such as high-speed running (HSR) to monitor training and match activities [1, 2]. This is of importance and relevance to practitioners to more appropriately prescribe training stimuli given the highly specified playing position roles of professional rugby league players. Typical rugby playing positions consist of ‘outside backs’, tasked with running at higher speeds during kick chase and kick return activities on the lateral areas of the field [3]; ‘adjustable’s’ who are required to run at higher speeds into open spaces whilst supporting play [4]; ‘wide-running forwards’ who are involved in ball carrying and tackling on the lateral areas of the field; and ‘hit-up forwards’ whose role it is to carry the ball through the middle of the field to assist in invading the opponents half of the field [5]. Of these playing positions it is the hit-up (106 ± 5 kg) and wide running forwards (99 ± 7 kg) who typically have higher mean body mass than the outside backs (96 ±4 kg) and adjustable’s (86 ± 8 kg) [6]. Such anthropometrical and tactical variations have been shown to influence the metabolic cost and locomotor characteristics of players during match-play [5], with outside backs (583m), adjustable’s (436m) and wide running forwards (418m) performing greater HSR distances than hit-up forwards (235m) [7].

A recent survey of practitioner applications and perceptions of HSR reported that 52% of rugby league practitioners apply absolute HSR thresholds among National Rugby League and European Super League teams, with the other 48% implementing individualised methods [8]. According to the literature, the most recently applied absolute HSR threshold is 5.5 m·s^-1^ [2, 8, 9], where the same speed is applied to all players. However, this contradicts thresholds of 5.0 m·s^-1^ which have been commonly used within the scientific literature [10]. Practitioners who individualised HSR have demonstrated preference for implementing peak sprint speed methods (*n = 9*), whereby practitioners measure players’ peak sprint speed and quantify HSR as a standardised percentage of the speed achieved. Maximal aerobic speed (MAS) methods (*n = 2*) were also reported [8], with these methods anchoring HSR to a speed achieved during a fitness-based test. However, none of the individualised methods reported by Bennett et al [8] are present in previously published rugby league research.

Recent literature adopted a data-mining approach and applied it to microtechnology data from sixteen teams during National Rugby League match-play to assist in the standardisation of velocity zones in rugby league. Cummins et al [11] stated an absolute HSR threshold of 5.8 m·s^-1^ should be used to analyse the external loads of elite male rugby league players [11]. This HSR threshold exceeds the threshold of 5.5 m·s^-1^ which has previously been implemented in league-wide studies in an attempt to produce broader data sets [1, 2]. This study suggests that current absolute thresholds may well under report the volume of HSR and the aerobic fitness requirements of elite rugby league players [10, 11]. However, the approach applied within this study is purely statistical and has no direct link to physiology or physical adaptation. With this in mind, absolute HSR thresholds will differ from individualised HSR thresholds derived from laboratory [12] or field-based tests [13], which will result in a conflicting interpretation of HSR data [8]. Practitioners who favour HSR thresholds derived from a form of fitness testing need to ensure that the test prescribed is practical and has previously been documented to be physiologically valid.

Quantifying HSR using speeds derived from physiological-based assessments that quantify the second ventilatory threshold (VT^2^) and MAS have previously been reported [13, 14]. VT^2^ corresponds to the inflection in the ventilatory equivalents for both oxygen and carbon dioxide, whilst there is a corresponding reduction in the end tidal pressure of carbon dioxide [15]. MAS is defined as the lowest running speed (m·s^-1^) at which $\dot{V}O_{2\max}$ occurs [16], and it has previously been suggested as a well-defined metric suitable for identifying relative exercise intensity and physiological adaptation [17]. More recently, MAS methods have been suggested to give practitioners a more practical insight into the time and distance above a speed associated to players’ physiology [10, 17, 18]. Despite the intermittent nature of rugby league having an increase in anaerobic energy demand, running at high-intensity aerobic speeds can be seen as crucial to develop the maximal aerobic power of players [19] With MAS values differing between rugby league players [20], practitioners aim to achieve physiological adaptation by implementing MAS to individualise the stimulus of high-intensity conditioning [21]. It has been well documented that aerobic capacity is a pivotal characteristic of rugby league players [22, 23], suggesting that MAS methods may also be implemented to individualise traditional speed zones within rugby league [10].

Individualising speed zones using methods that quantify MAS such as an incremental treadmill test or a distance-based time trial have previously been adopted in soccer [15, 18]. MAS testing procedures such as a 5-minute run or 1200 m shuttle test (1200m ST) have been prescribed by rugby league practitioners to quantify HSR [8], despite not being previously validated within the literature. This may be due to these field-based methods being more practical, whereby its more time and cost efficient to test multiple players at once then criterion measures for MAS such as an incremental treadmill test [8, 10]. However, characteristics of both the field-based tests mentioned differ, with the 5-minute run being continuous and linear and the 1200m ST being continuous and shuttle based. This may propose the 1200m ST to have an increased metabolic energy contribution, although more ecologically valid due to being more related to the sport practiced [24]. Despite this, it remains unclear if the field-based tests mentioned accurately quantify MAS, as under and/or over estimations of MAS can subsequently lead to misinterpretation of HSR data. Therefore, the aim of this study was to validate a 5-minute time trial and 1200m ST to determine MAS, which can be implemented by practitioners to quantify HSR in rugby league.

## Methods

### Subjects

Ethical approval was granted through the Faculty of Health Sciences at the University of Hull (FHS:22–23.26). Players agreed to participate after reading a participant information sheet, by signing informed consent forms in paper format and completing an institutional pre-exercise medical questionnaire. Twenty first team professional rugby league players (Age: 23.1 ± 4.7 y, Body Mass: 95.4 ± 7.7 kg, Height: 182.9 ± 5.4 cm) for a team competing in the Betfred Super League participated in this study. Players were categorised positionally as outside backs (n = 5), adjustable’s (n = 5), wide-running forwards (n = 5) and hit up forwards (n = 5). Data was collected over a 2-week period during the latter stages of the scheduled pre-season period (Jan 2023) (Table 1). The sample size attained within this study (n = 20) was the maximum number of players available to complete all trials during the testing phase, which is similar to sample sizes implemented in previous rugby league-based research [21, 25–31].

**Table 1:** Descriptive statistics, tests for differences and correlation analyses for physiological variables collected during the three different trials in professional rugby league players.

Descriptive statistics				Tests for differences			Pearson correlation coefficient	
	Incremental Treadmill Test	5 Minute Run	1200m ST	RM ANOVA F(df), p	p(bonf) Incremental Treadmill Test v 5 Minute Run	p(bonf) Incremental Treadmill Test v 1200m ST	r, p Incremental Treadmill Test v 5 Minute Run	r, p Incremental Treadmill Test v 5 Minute Run
MAS (m·s^-1^)	4.8 ± 0.4	4.3 ± 0.3	4.0 ± 0.2	F(_1.244, 23.630_) = 120.005, p< 0.01)	p < 0.001	p < 0.001	0.64, p = 0.02	0.70, p<0.001
HR_max_ (bpm)	188 ± 8	182 ± 8	185 ± 7	F(_2,38_) = 11.64, p<0.001)	p < 0.001	p = 0.18	0.70, p<0.001	0.73, p<0.001
La_peak_ (mmol)	11.7 ± 2.5	9.6 ± 1.2	13.8 ± 1.7	F(_1.53,29.13_) = 35.15, p<0.001)	p < 0.001	p < 0.001	0.03, p = 0.90	0.53, p = 0.53
RPE (AU)	9.1 ± 1.0	9.1 ± 0.8	9.2 ± 0.7	F(_2,38_) = 0.15, P = 0.86)	-	-	0.25, p = 0.28	0.56, p = 0.01

Key: Maximal Aerobic Speed (MAS), Meters per second (m·s^-1^), Peak Heart Rate (HR_peak_), Beats per minute (bpm), Peak Lactate (La_peak_), Millimole (mmol), Rating of Perceived Exertion (RPE), Arbitrary Units (AU), Degrees of Freedom (df), Bonferroni (bonf), Repeated Measures (RM).

### Design

**Fig 1:**
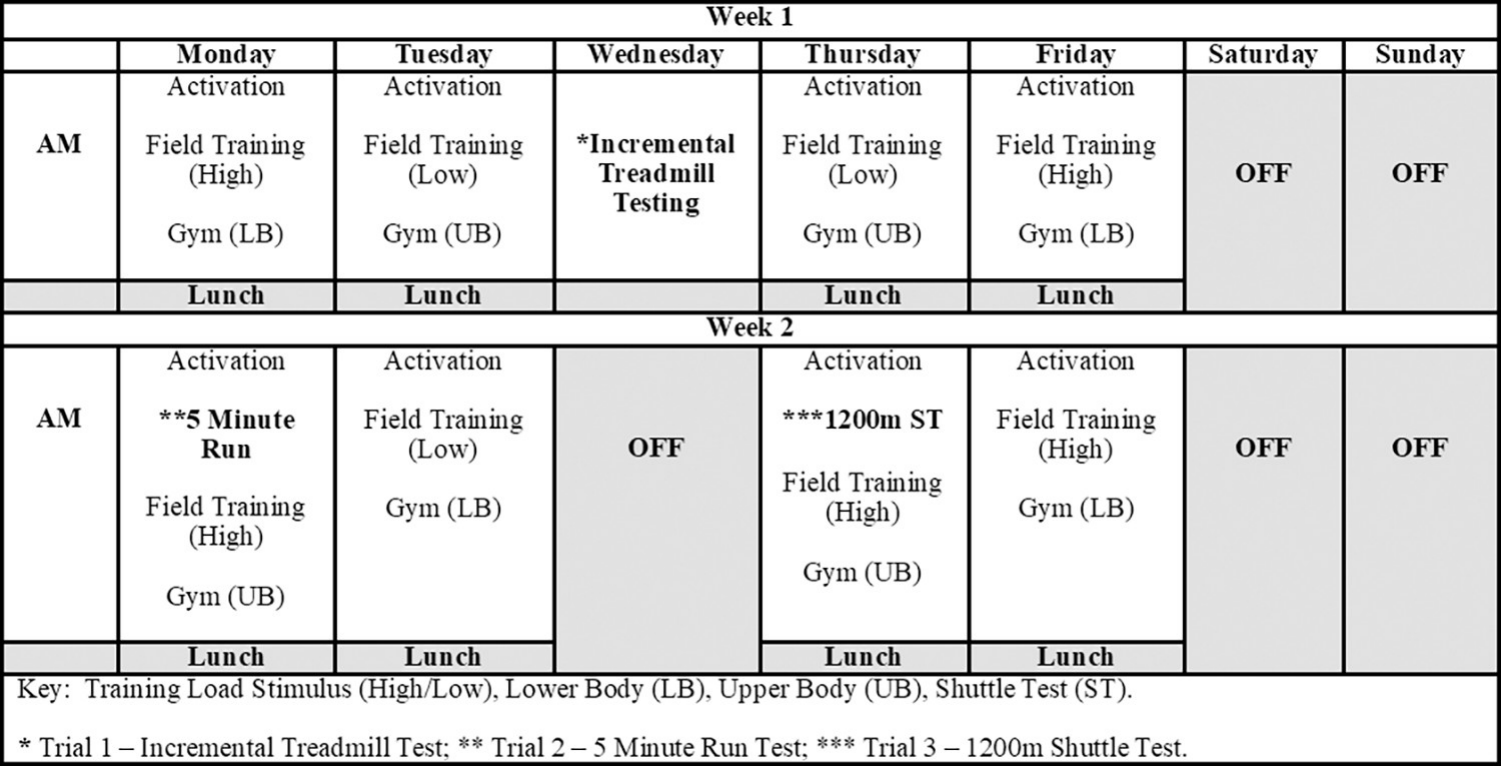
Periodization. Periodization of the two-week training period which included the three testing trials.

A repeated measures study design was implemented were each player performed one trial per visit on three separate occasions. Players continued their normal team training procedures during a two-week period as scheduled by the club coaching staff (Fig 1) This two-week training period included eight training days (e.g., pitch and gym-based work), one testing day and five recovery days (testing commenced 4^th^ January 2023 and was completed on the 12^th^ January 2023). All testing was completed within this period due to the limited availability of staff and the facilities required. All players were required to provide subjective wellness data (as per normal training procedures) to ensure they were in a non-fatigued state before testing commenced. Prior to study inclusion, the club’s medical staff screened players to identify if they were free from any form of illness or injury.

### Trial 1: Laboratory treadmill test

Players where required to attend the laboratory at least 24 hours after their previous training session (Fig 1). Players were advised to continue with their normal dietary requirements but were advised not to consume caffeine in the three hours before the test commenced to prevent any performance enhancement [32]. During this visit, players individually completed an incremental treadmill test to determine the speed at maximal exhaustion. The motorised treadmill (Pulsar Cosmos, Sports & Medical, Nussdorf-Traunstein, Germany) had previously been calibrated and started at a warm up speed of 7 kmh^-1^ for 3 minutes and then proceeded to increase by 0.2 kmh^-1^ every 12s until maximal exhaustion [15]. Players were blinded to the treadmill speed to ensure a maximal test was performed and were encouraged both before and during the test to perform maximally. The speed at the time of exhaustion was collected as MAS [15, 16, 33].

### Trial 2: 5 minute run

**Fig 2:**
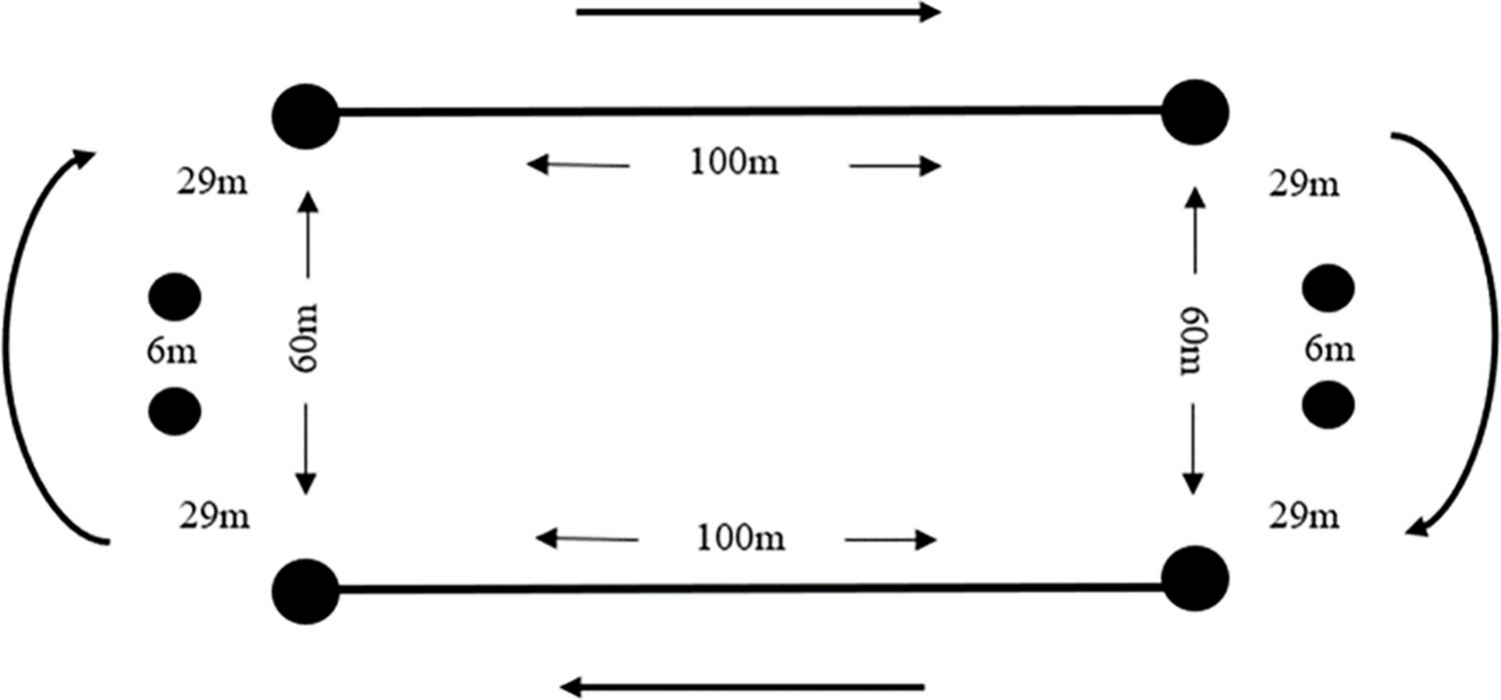
5-Minute run. Configuration of the 5-minute run.

Five days after trial one players completed a 5-minute, running time-trial. Players participated in a standardised warm up before completing the trial. Players completed the time trial in groups of five based on playing position and were positioned individually around the track before the test commenced. A linear running track was marked out on an artificial 4G surface and players were instructed to run around the track (modified rugby pitch dimensions) in a clockwise direction and encouraged to achieve the maximal distance possible in the available time (Fig 2). Audio signals notified players after every completed minute [33] and players were instructed to remain stationary at the completion of the test. MAS was calculated as the total distance covered during the test divided by the test duration in seconds (e.g., 1500m/300s = 5.0 m∙s^-1^) [19]. The total distance covered was quantified using the Catapult Vector S7 GPS units [34] and Catapult Openfield Software (Catapult Sports, Melbourne, Australia).

### Trial 3: 1200m shuttle test

**Fig 3:**
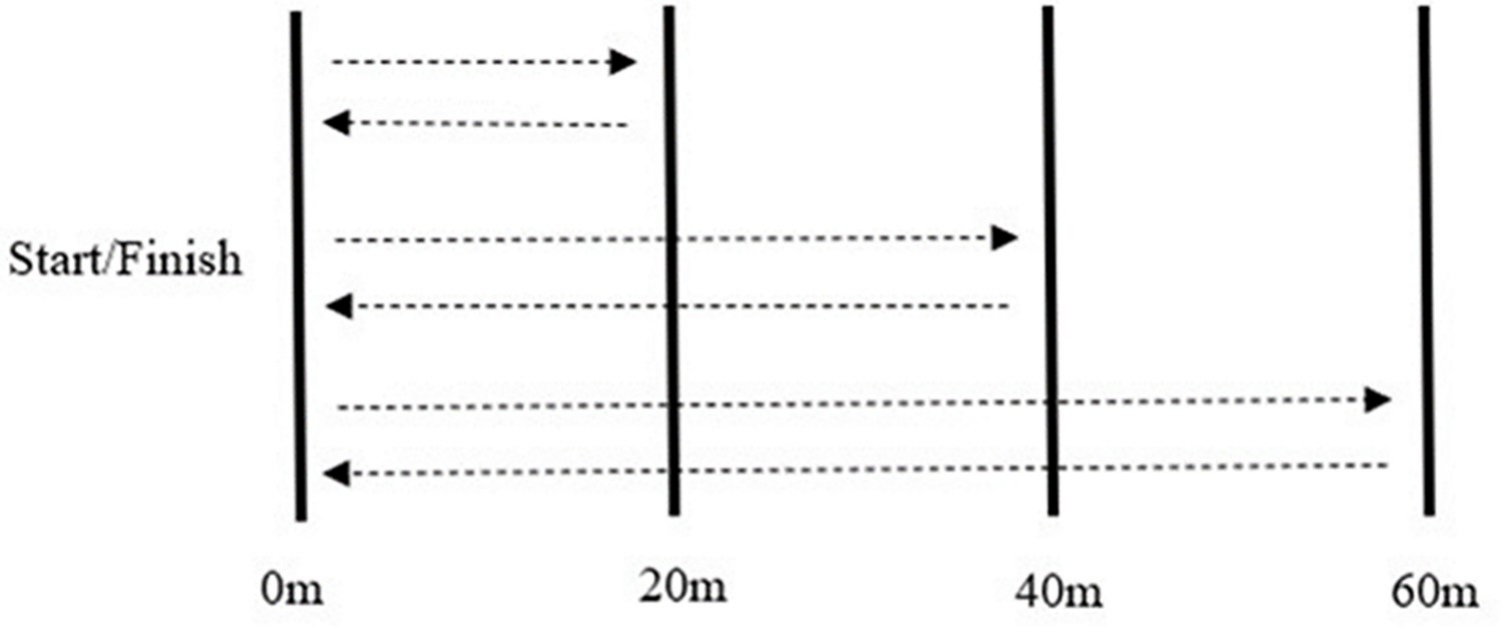
1200m ST. Configuration of the 1200m Shuttle Test.

Three days after trial two, players completed the 1200m ST. Players were required to participate in a standardised warm up before completing the test. Players completed the test in groups of five based on playing position and were positioned five meters apart on the start line. A linear running track was marked out on an artificial 4G surface and players were required to perform one set of continuous shuttle runs (20m and back, 40m and back, 60m and back) five times [35–38] (Fig 3). Players were encouraged to run the test at maximal effort to achieve the shortest time possible to complete the test, with sound signals notifying players after every completed minute. The time taken to complete the test in seconds was derived for each player using a stopwatch. This time in seconds was then used to calculate MAS (e.g.,1200m/300s = 4.0 m∙s^-1^) [35].

### Physiological measurements

#### Maximal aerobic speed

During all trials, each player was required to wear a Catapult Vector GPS sports vest with integrated heart rate accompanied by a 10 Hz GPS unit positioned between the scapulae (Vector S7, Catapult Sports, Melbourne, Australia). The speed achieved at the termination of the incremental treadmill test was collected as MAS. The distance covered and time to complete was collected during both trial two and three to calculate MAS using the formulas mentioned above, in each trial section, respectively.

#### Heart rate

Peak heart rate (*HR*_*max*_) was quantified upon immediate completion of the trials through the Catapult Vector integrated heart rate vest and GPS unit. HR_max_ was identified using the Catapult Openfield software (Catapult Sports, Melbourne, Australia).

#### Blood lactate

Blood lactate (*La*) was also collected one and three minutes post completion of each trial [39, 40] (Lactate Plus, Nova Biomedical, USA) [41] with each player providing one finger-pick blood sample at each time point and the highest of these two values being identified as (La_*peak*_).

#### Rating of perceived exertion

The rating of perceived exertion (RPE) was collected upon completion of each trial using CR10 scale [42].

### Statistical analysis

Statistical analysis was completed using JASP software (JASP Team 2023 Version 0.17. 1). The mean and standard deviation were calculated to represent descriptive statistics for all variables. Data were tested for sphericity using Mauchly’s test of sphericity and corrected with Greenhouse-Geisser correction as appropriate. Difference across trials were analysed using a repeated measures ANOVA for each physiological variable. Where a significant difference was displayed (p<0.05), pairwise post-hoc testing with a Bonferroni correction was utilised. The strength of relationships between trials was determined by applying Pearson’s correlation coefficient to each of the collected variables during the three trials. Correlation values established relationships between trials as, *small* (r = 0.1–0.3), *moderate* (r = 0.3–0.5), *large* (r = 0.5–0.7), *very large* (r = 0.7–0.9) *and almost perfect* (r = 0.9–1.0) [43]. Bland-Altman plots were conducted to assess the magnitude of difference between the variables for all trials to establish the level of agreement between data sets [44].

## Results

### Maximal aerobic speed

**Fig 4:**
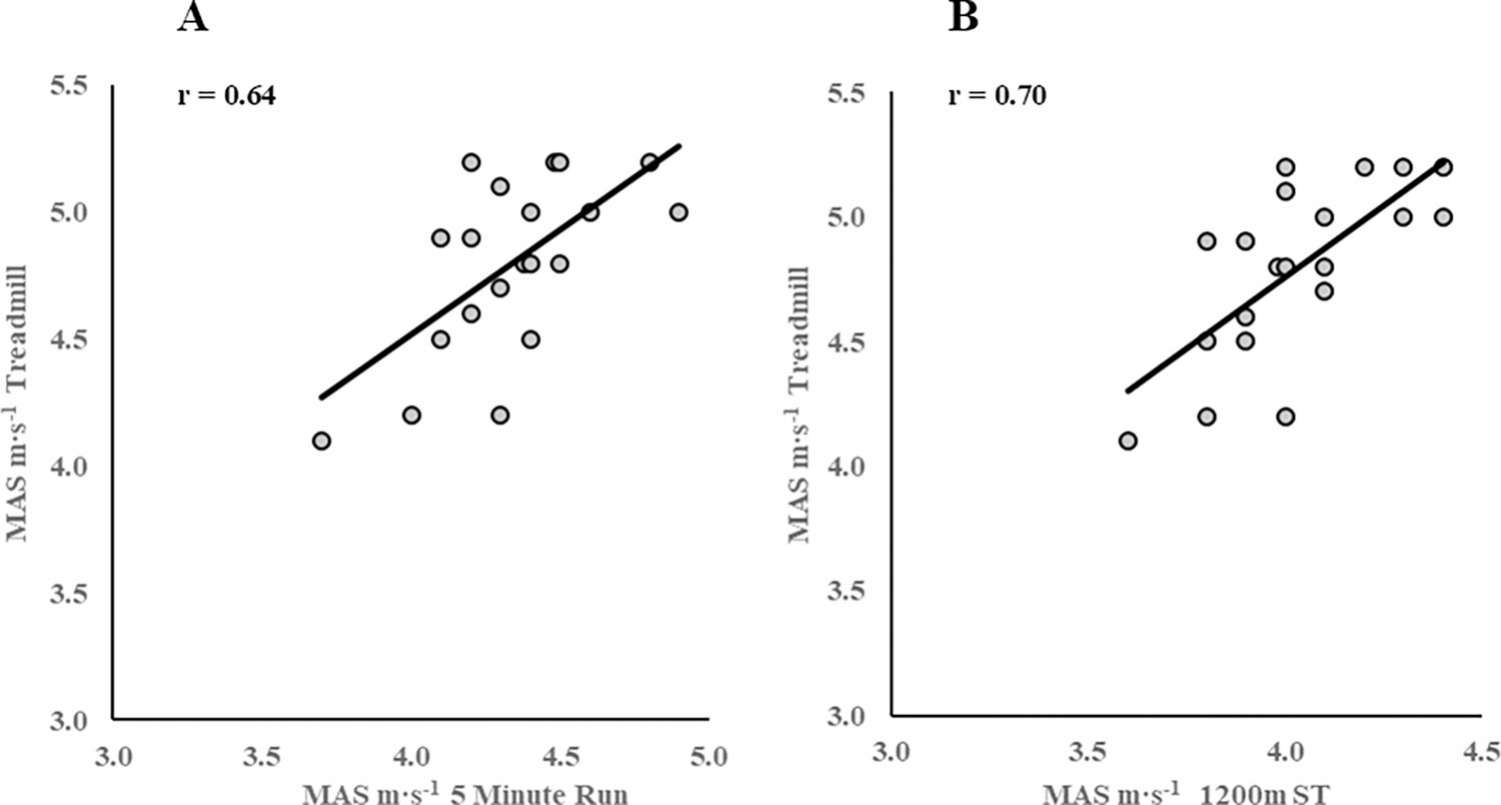
Correlations for MAS. Represents scatterplots showing the Pearson’s correlation coefficient of MAS derived from the incremental treadmill test and 5-minute run (**4A)** and the incremental treadmill test and the 1200m ST (**4B**).

**Fig 5:**
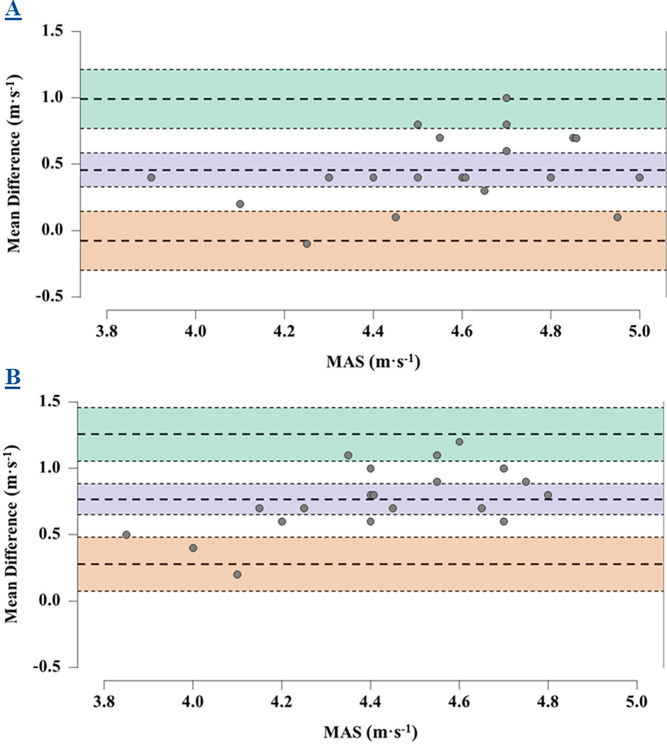
Mean Differences for MAS. Bland-Altman plots demonstrating the mean difference with 95% confidence intervals (lilac shaded area) and subsequent level of agreement for MAS for the incremental treadmill test and 5-minute run (**5A**) and the incremental treadmill test and 1200m ST (**5B**). The mean difference + 1.96 standard deviation (green shaded area) and mean difference– 1.96 standard deviation (orange shaded area) with upper and lower confidence intervals are also shown.

Of the three trials completed by players, MAS was significantly higher during the incremental treadmill test (4.8 ± 0.4 m·s^-1^) when compared to the 5-minute run (4.3 ± 0.3 m·s^-1^, p = <0.001) and 1200m ST (4.0 ± 0.2 m·s^-1^, p = <0.001) (Table 1) The 5-minute run (r = 0.64) and 1200m ST (r = 0.70) were both shown to have large correlations for MAS when compared to the incremental treadmill test (Fig 4). The magnitude of mean difference for MAS was the lowest for the 5-minute run (0.45 m·s^-1^) when compared to the 1200m ST (0.75 m·s^-1^) (Fig 5).

### Heart rate

During the trials, *HR*_*max*_ was significantly higher during the incremental treadmill test (188 ± 8 bpm) when compared to the 5-minuite run (182 ± 8 bpm, p = <0.001) although no significant differences were present for the 1200m ST (185 ± 7 bpm, p = 0.18) (Table 1). The 1200m ST demonstrated to have a very large correlation for *HR*_*max*_ with the incremental treadmill test (r = 0.73) (Table 1). The magnitude of mean difference for *HR*_*max*_ was the lowest for the 1200m shuttle test (2 bpm) (Table 2).

**Table 2:** Represents the mean differences with upper and lower confidence intervals for all physiological variables collected during the field-based running trials when compared to the incremental treadmill test in professional rugby league players.

Incremental Treadmill Test			Mean Difference			Mean Difference + 1.96SD			Mean Difference– 1.96SD		
		Mean Diff	% Diff *	Lower CI	Upper CI	Mean Diff	Lower CI	Upper CI	Mean Diff	Lower CI	Upper CI
	**5 Minute Run**										
MAS		0.45	-9.38	0.33	0.58	0.99	0.77	1.21	-0.08	-0.30	0.14
HR_peak_		5.60	-2.98	2.84	8.36	17.14	12.37	21.91	-5.94	-10.71	-1.17
La_peak_		2.02	-17.26	0.76	3.28	7.29	5.11	9.47	-3.25	-5.43	-1.07
RPE		0.00	0.00	-0.53	0.53	2.20	1.29	3.11	-2.20	-3.11	-1.29
	**1200m ST**										
MAS		0.77	-16.04	0.65	0.88	1.25	1.05	1.46	0.28	0.07	0.48
HR_peak_		2.25	-1.20	-0.35	4.85	13.15	8.64	17.66	-8.65	-13.16	-4.14
La_peak_		-2.09	17.86	-3.09	-1.10	2.09	0.36	3.82	-6.28	-8.01	-4.55
RPE		-0.10	1.10	-0.50	0.30	1.57	0.88	2.26	-1.77	-2.46	-1.08

Key: Maximal Aerobic Speed (MAS), Peak Heart Rate (HR_peak)_, Peak Lactate (La_peak_), Rating of Perceived Exertion (RPE), 1200m Shuttle Test (1200m ST), Confidence Interval (CI), Standard Deviation (SD)

*Represents the difference as a percentage when compared to the incremental treadmill test values.

### Blood lactate

In regard to La_*peak*,_ the incremental treadmill test (11.7 ± 2.5 mmol) had significantly lower values than the 1200m ST (13.8 ± 1.7 mmol, p = <0.001), with the incremental treadmill test having significantly higher values than the 5-minute run (9.6 ± 1.2 mmol, p = <0.001) (Table 1). However, the 5- minute run demonstrated a very large correlation for La_*peak*_ with the incremental treadmill test (r = 0.90) (Table 1). The magnitude of mean difference for La_*peak*_ was the lowest for the 5-minute run (2.0 mmol) (Table 2).

### Rating of perceived exertion

The incremental treadmill test (9.1 ± 1.0 AU) displayed no significant differences for RPE when compared to the 1200m ST (9.2 ± 0.7 AU), with no significant differences also present between the incremental treadmill test and 5-minute run (9.1 ± 0.8 AU) (Table 1). The 1200m ST was shown to have a large correlation for RPE with the incremental treadmill test (r = 0.56) (Table 1). When compared to the incremental treadmill test, the 5-minute run produced equal mean values for RPE (Table 2).

## Discussion

The purpose of this study was to examine the validity of two field-based tests (5-minute run and 1200m ST) to quantify MAS in comparison to a gold standard measure. The main finding demonstrates that MAS derived from the 5-minute run has a greater level of agreement when compared to MAS from the 1200m ST. The study also demonstrates that physiological variables such as La_peak_ and RPE quantified during the 5-minute run also have a greater level of agreement when compared with these variables determined during 1200m ST. These findings may propose the 5-minute run to be a more valid field test to quantify MAS.

The current study reports the 5-minute run and 1200m ST to both have large correlations for MAS when compared to MAS quantified during an incremental treadmill test (Table 1; Fig 4). Normative MAS data reviewed previously among team sports athletes has suggested the 5-minute run to be a valid measure of MAS [16, 20]. As well as valid, the 5-minute run has also been documented to be a reliable measure of MAS among semi-elite rugby union players (intraclass correlation coefficient: 0.98) [45]. Further, research conducted by Berthon et al [16, 33] quantified MAS during a 5-minute time trial and compared it to an incremental treadmill during two studies of athletes of varying fitness levels. These studies highlighted an almost perfect relationship and a very large correlation for MAS between the two tests, with the correlation greater than that of the current study respectively (r = 0.94) [16] (r = 0.86) [33]. However, within the current study, the mean MAS was significantly higher for the treadmill (p = <0.001) in comparison to the 5-minute run (5-minute run: 4.3 m·s^-1^, treadmill: 4.8 m·s^-1^) (Table 1). However, Berthon et al [16, 33] expressed a higher value for MAS from the 5-minute run in both studies (5-minute run: 4.8 m·s^-1^, treadmill: 4.7 m·s^-1^) [16], (runners: 5-minute run: 5.42 m·s^-1^, treadmill: 5.38 m·s^-1^, non-runners: 5-minute run: 4.42 m·s^-1^, treadmill: 4.36 m·s^-1^) [33]. It could be suggested that the difference identified between those studies and the current study is a result of not only the population of athletes but also how familiar the athletes were with the test, the different methodologies implemented, and the formulas applied to calculate MAS. However, in relation to the current study, it could be suggested that the 5-minute run provides a sound physiological rationale due to the MAS achieved being associated to the duration of the test. It’s critical that the test duration is necessary to elicit the maximal aerobic component with a reduced anaerobic contribution [16]. The 5-minute run results in fatigue, but this fatigue does not exceed a certain limit, as MAS calculated from running performance is related to the intersection of anaerobic and aerobic lines which has found the optimal duration for a MAS measurement to be 4.97 minutes [16]. This offers a reasonable explanation in the current study as to why the 5-minute run may be a more physiologically sound test to quantify MAS than the 1200m ST. Moreover, it could be proposed that with advancements in technology, the methods and formulas used within the current study maybe preferred by rugby league practitioners to quantify MAS, due to the current study population being current professional rugby league players.

Previously, MAS derived from the 1200m ST has never been compared to MAS derived from an incremental treadmill test across all sports, although the current study demonstrates the 1200m ST to have a very large correlation when compared (Table 1). However, literature documents the mean speed obtained during the test has been compared to the speed obtained during the 30:15 Intermittent Fitness Test (r = 0.73) [35] highlighting a very large correlation among semi-elite rugby league players. Although this was the case, the MAS values derived during the 1200m ST within the study by Kelly et al [35] are lower than those found in the current study (Kelly et al: 3.6 ± 0.3 m·s^-1^, current study: 4.0 ± 0.2 m·s^-1^). It could be suggested that a reasoning for this is due to the professional training status and enhanced fitness levels of the players included in the current study. Moreover, the MAS values in the current study derived from the 1200m ST are higher than those previously achieved during an alternative shuttle based running test (Multi-Stage Shuttle Test) conducted by Berthoin et al [46] (current study: 4.0 ± 0.2 m·s^-1^, Berthoin et al [46]: 3.6 ± 1.0 m·s^-1^. This study established that the requirement to accelerate and decelerate in combination with individual body mass will negatively influence MAS during shuttle-based running. In support of this, it has previously been suggested that the 1200m ST may underestimate MAS due to the requirement for players to decelerate, turn and accelerate during shuttles and in order to estimate MAS, it proposed a correction factor of 1.3s per turn, i.e., 37.7s should be subtracted from the completion time [36]. However, this has not been validated and although this may attempt to correct MAS for the 1200m ST, it doesn’t take into consideration each player’s physical characteristics (i.e., body mass) and their individual ability to turn efficiently [36]. In relation to the current study, the studies mentioned support that the 1200m ST underestimates MAS despite its strong correlation with the incremental treadmill test within the current study.

Although the 5-minute run and 1200m ST have large and very large correlations for MAS, this only takes into consideration the strength of the relationship between MAS data and doesn’t consider the level of agreement. Fig 5 shows the mean difference between the 5-minute run and 1200m ST with the incremental treadmill test within a Bland-Altman plot. It is evident within this study that the 5-minute run has a higher level of agreement when determining MAS due to a lower mean difference in comparison to the 1200m ST. It could be suggested that despite the 1200m ST having a stronger correlation for MAS, the higher level of agreement for the 5-minute run would propose this test to be more valid. Nevertheless, the 1200m ST possesses greater ecological validity than the 5-minute run due to the inclusion of acceleration, deceleration and change of direction movements similar to those experienced during rugby league match-play. However, as a result of this, lower values of MAS are quantified during the 1200m ST which in turn reduces the construct validity for this test. Conversely, the 5-minute run despite having lower ecological validity due to its continuous and linear nature which is unlike rugby league match-play movements, quantifies MAS values which are in closer proximity to those derived from the incremental treadmill test, therefore proposing an increased construct validity for this test. However, practitioners may question the tests practical acceptability to quantify MAS, due to it underestimating MAS by 0.45 m·s^-1^ (Table 2). That said, it needs to be considered that the underestimation of MAS from the 5-minute run may be due to an overestimation of MAS from the incremental treadmill test. This could be due to the speed at volatile exhaustion within the current study being derived as MAS instead of the minimum speed at which elicited $\dot{V}O_{2\max}$. Despite both field-based tests underreporting MAS, a correction equation could be applied to the two field-based tests to estimate MAS more accurately, although consideration needs to be taken into the physiological response of the tests included in this study to better determine their validity.

Maximum heart rate measured in the current study resulted in very large correlations for both the 5-minute run and 1200m ST respectively (Table 1). However, the level of agreement for HR_max_ was higher for the 1200m ST (Table 2), although the HR_max_ values during all trials during the current study were lower than those reported previously during an incremental treadmill test and 5-minute run [16, 33]. The current study expresses HR_max_ to be significantly higher during the treadmill test than the 5-minute run, but not significantly higher than the 1200m ST (5-minute run: p = <0.001, 1200m ST: p = 0.18), with HR_max_ during the 5-minute run to be the lowest (Table 1) and all HR_max_ values greater than HR values typically reported during rugby league match-play [47]. Previous studies support HR_max_ resulting in marginally lower values for the 5-minute run when compared to the treadmill test in males of varied fitness levels (Treadmill: 192.0 ± 7.5 bpm, 5-minute run: 191.8 ± 8.1 bpm) [16] and in elite runners, (Treadmill: 191.3 ± 7.3 bpm, 5-minute run: 189.4 ± 7.3 bpm) [33] although this was not the case for non-runners (Treadmill: 194.3 ± 7.0 bpm, 5-minute run: 194.7 ± 8.1 bpm) [33]. A key observation from the field-based tests in the current study report that La_peak_ has a large correlation for the 1200m ST (Table 1) when compared to the treadmill, with La_peak_ exceeding the values identified in the treadmill test (Table 1). However, in terms of mean difference, La_peak_ achieved in the 5-minute run has a higher level of agreement with La_peak_ during the treadmill (Table 2), due to La_peak_ during the 1200m ST significantly exceeding the treadmill values (p = <0.001), consequently resulting in a negative mean difference. The values reported in this study (9.6 ± 1.2 mmol) are similar to those previously reported by Berthon et al (9.9 ± 2.1 mmol) [16] (9.9 ± 1.7 mmol) (9.5 ± 2.2 mmol) [33] for the 5-minute run. However, this literature documents La_peak_ to be higher during the 5-minute run which is inconsistent with the findings of the current study. Values of La_peak_ achieved in the 5-minute run could be considered similar to values that have typically been reported during rugby league match-play [47].

The current study’s findings along with previous findings may suggest that athletes with a higher training status (i.e., professional rugby league players) may elicit a higher HR_max_ when performing the treadmill test in comparison to the 5-minute run and 1200m ST. That said, current team sport literature does not document HR_max_ or La_peak_ values achieved during the 1200m ST in order to compare previous data, proposing this study to be the first to do so. With the 1200m ST having a lower mean difference and therefore a higher level of agreement for HR_max_ as well as an increased lactate contribution, it could be suggested that HR_max_ and La_peak_ values are elevated during the 1200m ST, due to a higher metabolic cost stemming from players being required to continuously accelerate, decelerate and change direction [10, 46]. Moreover, HR peaking during the treadmill test can be a result of increases in HR when running at faster speeds [48]. The lower HR_max_ and La_peak_ values for the 5-minute run observed within this study could be a result of this test being self-paced and linear, even though it has a similar mean test duration to the 1200m ST (5-minute run: 300.0 seconds, 1200m ST: 298.3 seconds), proposing physiological responses need to be considered when quantifying MAS using the field-based tests included within this study.

Rating of perceived exertion has never been measured during previous validation studies such as Berthon et al [16]. However, the current study demonstrates no significant differences for RPE values when collected on completion of the 1200m ST in comparison to the incremental treadmill test (Table 1). Not only are they higher for the 1200m ST, but also a large correlation is present when compared to the treadmill. Nevertheless, the level of agreement between tests for RPE is higher for the 5-minute run, due to a lower mean difference. It could be suggested that the higher values reported for the 1200m ST, may be due to the mechanical demand of the test and its association to an increased metabolic cost and subsequent increased HR_max_ and La_peak_ values. Moreover, implementing a differential RPE may have been a better option to differentiate the 1200m ST from the other two tests.

The current study provides rugby league practitioners with important information regarding the prescription of two field-based tests to quantify MAS. However, this study is not without its limitations. Firstly, the incremental treadmill test was conducted individually, while the field-based tests were conducted simultaneously with other players. Test performance during the field-based tests may have been affected due to reduced or enhanced motivation of performing the test alongside other players which may have been alleviated by all players completing these tests individually. Secondly, the speed at the termination of the treadmill test was quantified as MAS as opposed to the speed at which maximal oxygen consumption first occurred, leading to MAS data potentially being overestimated for this test. Other limitations of the current study include no disclosure of any reliability data for the collected measurements, as well as oxygen consumption data to demonstrate the differing energy contribution between tests also being absent.

## Practical applications

This study establishes the 5-minute run to be a more valid method to quantify MAS when compared to the 1200m ST. Practitioners should not compare MAS derived from the 5-minute run and 1200m ST and should progress with the same test longitudinally. For the purposes of conditioning and the individualisation of HSR thresholds, practitioners should understand that both field-based tests in this study underreport MAS. Future research should assess how the individualisation of HSR using MAS methods in the current study change the interpretation of HSR data during match-play and training practice.

## Conclusion

To conclude, this study establishes the 1200m ST to have a stronger relationship for MAS than the 5-minute run when compared to a gold standard measure, however the 5-minute run results in a smaller mean difference and therefore a higher level of agreement for the determination of MAS. The metabolic demand of the 1200m ST results in physiological and psychophysiological variables such as La_peak_ and RPE exceeding values of the incremental treadmill test, suggesting a reason for the underestimation of MAS (0.77 m·s^-1^). The 5-minute run although self-paced, produces a more accurate measurement for MAS than the 1200m ST, despite an underestimation of 0.45 m·s^-1^. This study establishes the 5-minute run to be a more valid measure of MAS than the 1200m ST.

## Supporting information

S1 Dataset (XLSX)
